# Effects of core strength training combined with Tai Chi Chuan for the musculoskeletal system and cardiopulmonary function in older adults: A study protocol for a randomized controlled trial

**DOI:** 10.1097/MD.0000000000012024

**Published:** 2018-08-21

**Authors:** Xiaofei Jia, Cai Jiang, Jing Tao, Yinyan Li, Yu Zhou, Li-dian Chen

**Affiliations:** a Fujian University of Traditional Chinese Medicine, Fuzhou, China.; b Fujian Provincial Hospital, Fuzhou, China.; c Outpatient Department of the Affiliated Medical Hall of Fujian University of Traditional Chinese Medicine, Fuzhou, China.; d People's Hospital of Ningxia Hui Autonomous Region, Yinchuan, China.

**Keywords:** cardiopulmonary function, core strength training, musculoskeletal, older adults, Tai Chi Chuan

## Abstract

**Background:**

According to the national census, China has gradually become an aging society; moreover, aging has become an irreversible worldwide trend in the twenty-first century. Aging can lead to decreased physical function, mobility, cardiopulmonary function and quality of life (QOL). The feasibility and benefits of core strength training (CST) and Tai Chi Chuan (TCC) in older adults (including improving cardiovascular, musculoskeletal, and mental health outcomes) have been confirmed in previous studies. To date, these exercise programmes have not been systematically compared, and the potential benefits of their combined use have not been tested. The primary objective of this **s**tudy protocol is to evaluate the effects of CST compared to those of TCC on the function of the musculoskeletal system and the cardiopulmonary system in older adults.

The second objective is to test the effectiveness of combined physical training that incorporates CST and TCC.

**Methods:**

A randomized, single-blind, parallel-controlled trial will be conducted. Three hundred eighty-four participants who meet the eligibility criteria will be randomly allocated into a control group, a CST group, a TCC group, and a combined group in a 1:1:1:1 ratio. Participants in the CST group and the TCC group will respectively receive CST and TCC training at a frequency of 1 hour per day, 5 days per week, totally 12 weeks. Participants in the combined group will receive 30 minutes CST and 30 minutes TCC training per day, 5 days per week, totally 12 weeks. No specific exercises will be required of the participants in the control group. Both musculoskeletal and cardiopulmonary function outcomes, including bone density detection, balance and coordination ability, walking ability, pain visual analogue scale (VAS) score, fall risk assessment, activities of daily living, pulmonary function tests, color sonography and electrocardiogram, will be evaluated by blinded operators at baseline, 13 weeks and 25 weeks (follow-up period).

**Discussion:**

The results of this study protocol are expected to clarify the synergistic effect of CST and TCC training on musculoskeletal and cardiopulmonary function in older adults. Furthermore, these findings will confirm whether combined or exclusive CST and TCC training, is more effective at improving functional outcomes in the elderly.

**Trial registration:**

Chinese Clinical Trial Registry: ChiCTR-IOR-17010769. Registration date: March 3, 2017.

## 1 Introduction

According to the sixth national census report in China, 13.26% of the total population are 60 years of age or older and 8.87% of the total populations are 65 years of age or older.^[1]^ These numbers suggest that China is facing the challenges of an aging population, and aging has become an irreversible worldwide trend in the twenty-first century. Advancing age is related to considerable changes in mental and physical health, including a loss of muscle mass and weakened musculoskeletal function.^[2]^ Furthermore, The elderly are at a risk of weakening respiratory function, given the age-related decline in lung function and respiratory muscle strength, as well as the age-related onset and progression of cardiopulmonary disease.^[3]^ All these factors associated with aging can lead to impaired physical function and mobility and reduce quality of life (QOL).^[4]^

More and more studies have proved that regular exercise or physical activities are good for physical and psychological health outcomes.^[5,6]^

Moreover, physical activity can enhance the functioning of cardiovascular systems and reduce cardiovascular risk.^[7,8]^ Tai Chi Chuan (TCC) has been practiced in China, Japan, South Korea, and other Asian countries for hundreds of years, especially in the elderly. TCC is a traditional physical and mental training that is light to medium intensity, depending on training style, posture, and duration. A large number of previous studies have shown that TCC can improve the body's aerobic capacity, muscle efficacy,^[9]^ and psychological well-being. In addition, TCC is beneficial to reduce common cardiovascular risk factors, such as hypertension, diabetes, dyslipidemia, and depression.^[10,11]^

Strength training has recently become a hot topic in many clinical studies, including the elderly.^[12,13]^ There are many studies indicating that strength training can improve the mobility of the human body (e.g., increase gait speed and distance), functional tasks (e.g., standing and shifting), and basic daily life functions of the elderly.^[14,15]^ In many sports activities, people have been increasingly aware of the importance of the central core of the body for stabilization and power generation. “Core stability’” refers to the ability to control the position and movement of the trunk over the pelvis so as to make the best production, transfer, and control of force and motion to the terminal segment during the comprehensive movement. Rehabilitation should include the restoration of the core itself and the core as the base for extremity function.^[16,17]^ In a recent study, the researchers conducted a nine-week progressive core strength training (CST) study on the unstable surface among the elderly in the community. It was concluded that CST conducted on unstable surfaces is a feasible and effective exercise programme to alleviate age-related performance decline.^[18]^

As mentioned above, both methods of physical training have positive effects on the outcome of human health. However, it is difficult to draw firm conclusions because limitations or biases exist in most studies due to factors such as insufficient sample size or a lack of strict randomized design. In addition, there is no systematic comparison of the effects of TCC and CST, nor has the possibility of a synergistic effect of the two physical training combinations been evaluated. The aims of this study protocol are to evaluate the effects of CST compared to those of TCC on the outcomes of the musculoskeletal system and cardiopulmonary function in older adults and to test the synergistic effect of a combined physical training of CST and TCC.

## 2 Materials and methods

### 2.1 Study design and setting

This study is a randomized, single-blind, parallel-controlled trial. Participants will be randomly allocated into 4 groups:

(i) control group (CG): routine activities of daily living;
(ii) CST group: routine activities of daily living plus core strength training;
(iii) TCC group: routine activities of daily living plus TCC; and
(iv) combined training group (CTG): routine activities of daily living plus core strength training and Tai Chi Chuan.

**Figure 1:**
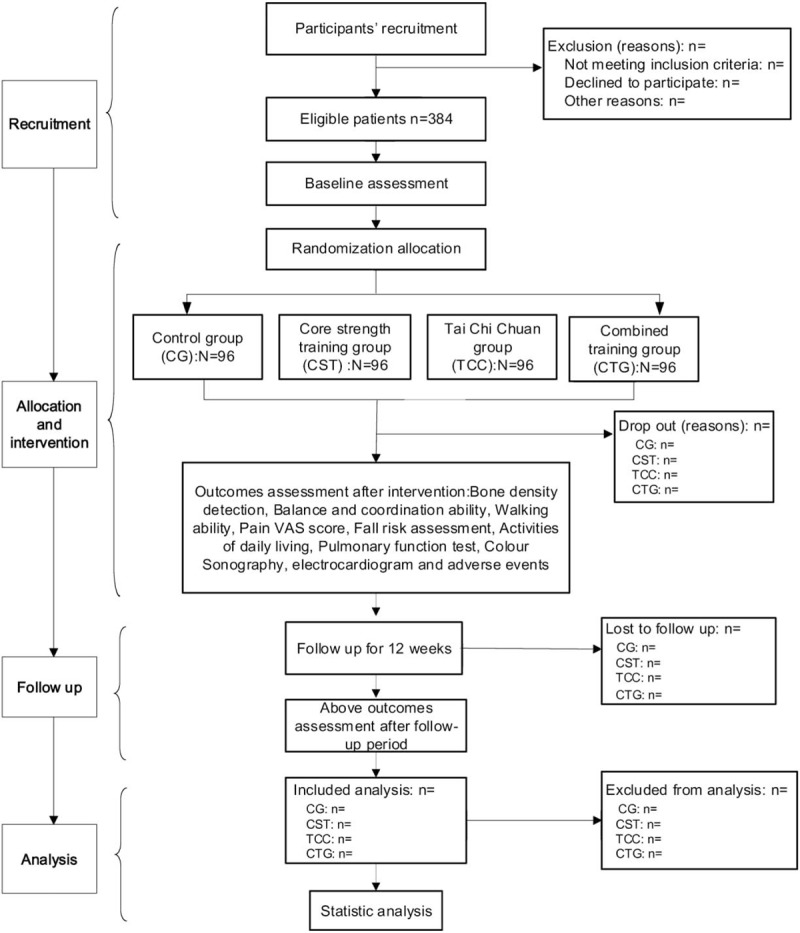
Flow diagram of participants.

All participants will receive the same intervention time of 1 hour/day, 5 days/week for a total of 12 weeks. Both musculoskeletal and cardiopulmonary function outcomes, including bone density detection, balance and co-ordination ability, walking ability, pain visual analogue scale (VAS) score, fall risk assessment, activities of daily living, pulmonary function test, color sonography, and electrocardiogram will be evaluated by the blinded assessors at the People's Hospital of Ningxia Hui Autonomous Region at baseline, at 13 weeks (end of the intervention), and at 25 weeks (12 weeks after follow up). At the end of each month during the intervention period, there will be professional trainers to guide the participants to ensure that the participants are trained correctly. The flow diagram of the entire trial programme is illustrated in Figure 1.

### 2.2 Sample size

We used change in bone mineral density (BMD) as the main effect indicator to estimate the sample size. Sample size calculations are performed to determine the number of participants necessary to detect effect sizes. Very few randomized controlled trials have examined the effect of TCC or CST on BMD in the elderly.

Based on similar previous studies^[19,20]^ and our preliminary experiment, the means with SDs of the BMD (lumbar spine) were X1 = 0.007 (SD1 = 0.098), X2 = 0.043 (SD2 = 0.108), X3 = 0.021 (SD3 = 0.0915), and X4 = 0.051 (SD4 = 0.099) in the CG, CST, TCC, and CTG, respectively. The sample sizes per group were calculated according to the formula: 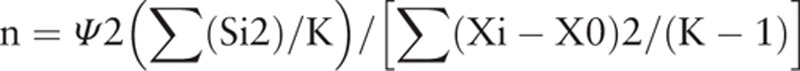

(i) with a type I error of 5% (α = 0.05) and 80% power (β = 0.10).
(ii) K: groups, K = 4.
(iii) Ψ: K = 4, V1 = K-1 = 3; V2 = N-1, N desirable maximum, Ψα, β, K-1,∞ = 2.17 (Obtained from statistical tables).
(iv) X0 = (X1 + X2 + X3 + X4)/K = 0.0305.

We can calculate the number of samples as n≈80, considering a 20% dropout and exit rate. Thus, a minimum of 384 total participants is necessary to reach the target of 96 participants per group.

### 2.3 Participants and recruitment

We will recruit 384 eligible participants from the Ningxia Yuehai Service Center. The eligible elderly must meet the following inclusion criteria. A CONSORT diagram of participant recruitment is shown in Table 1. We intend to promote the recruitment programme through advertisements on community bulletin boards and community broadcasts. Researchers will register the interested participants for the first time and conduct a corresponding screening assessment. After baseline assessment, eligible participants will be randomly assigned to 1 of the 4 research groups.

**Table 1:**
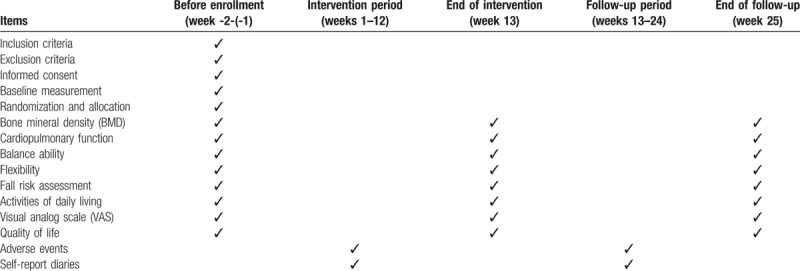
Trial processes chart.

### 2.4 Inclusion criteria

Participants must fulfill the following criteria:

(i) aged over 60 years;
(ii) no long-term regular exercise history, no long-term Tai Chi Chuan or core strength training participation;
(iii) no serious movement disorders, such as joint deformity, disease or severe spinal joint pain, severe heart and lung diseases, that would interfere with core strength training and Tai Chi Chuan;
(iv) no other serious organic diseases; and
(v) voluntary cooperation with the experiment of core strength training.

### 2.5 Exclusion criteria

Participants who meet1 of the following conditions will be considered ineligible:

(i) inability to train for an extended period time (this experiment requires 12 weeks); (ii) cognitive impairment;
(iii) severe mental illness; or
(iv) other active interventions that may affect the outcome of this study.

### 2.6 Withdrawal criteria

Participants who meet1 of the following conditions will be withdrawn from the trial:

(i) failure to train as required after the experiment;
(ii) suffer from bone or joint pain that impacts the continuation of training;
(iii) unable to continue training due to other diseases;
(iv) assigned to the control group but followed a regular exercise programme.

### 2.7 Randomization and allocation concealment

Following the baseline assessment, the subjects will be randomly assigned to1 of the 4 groups (CG, CST, TCC, and CTG) at a ratio of 1:1:1:1.

The randomization list will be generated through the Statistical Analysis Software (SAS, version 9.1) by a specified research assistant who is not otherwise involved in this study. We will use consecutive numbered, sealing and opaque envelopes to achieve the allocation concealment of the participants. The research manager will inform the eligible participants of the specific allocation results after the baseline assessment is completed.

### 2.8 Blinding

Due to the specificity of the intervention project, it is impossible to implement the blind method for participants and training instructors in this experiment. However, we will specify a designated research assistant to supervise the management of random allocation sequence and to blind coding the allocation by labelling the 4 groups (CG, CST, TCC, and CTG) A, B, C, and D. Furthermore, we will clarify the specific obligations of each investigator: the research assistant and training instructors will not be allowed to participate in the evaluation of outcomes; the outcome assessors and the statistical analysts will not be allowed to participate in screening and allocating. The participants will be told not to mention training forms in front of the assessors. We will uncover the allocation sequence and blind codes after the completion of the statistical analysis.

### 2.9 Interventions

#### 2.9.1 Control group

The subjects in the control group will maintain their original lifestyle and perform routine activities of daily living. During the intervention period of 12 weeks, if the participants change their daily activities or increase their amount of exercise or exercise intensity, they will be required to record the details of the extra physical activity, including the type, intensity, and activity time.

#### 2.9.2 Core strength training group

In addition to the routine activities of daily living, the participants in the CST group will receive 12 weeks of CST. The CST programme will be supervised and guided by 2 experienced physiotherapists who work in the Ningxia Hui Autonomous Region People's Hospital and will ensure that each participant is familiar with a standard CST programme. The core strength training programme consists of 5 core movements:

(i) Double leg bypass: in the supine position, the participants are required to flex the hip joint, flex the knee joint, and then use their back to raise their waist and buttocks and to maintain hip extension to increase the proprioception of the back;
(ii) Single leg bypass: in the supine position, the participants are required to flex the knee and hip of the left leg, keep the right leg straight, and then raise their lower back, hip, and right leg while maintaining bilateral hip extension in the air, using the shoulders and left leg only as a fulcrum, alternating twice on each side;
(iii) Supine step: in the supine position, the participants are required to take a bilateral leg knee flexion, alternating the left and right feet step by step, to increase hip balance;
(iv) Four-point support (abdominal bridge): in the prone position, the participants are required to use both elbows and feet as a fulcrum, with the body propped up and suspended in the air, facing front throughout the action;
(v) Kneeling diagonal support: the participants will take a kneeling position, supporting the body with both hands and knees, with the arms straight, both hips flexed and a knee flexion of 90 degrees; then the left side of the upper arm will point straight forward and the right leg will point backward, so that the body will be supported only with the right hand and left knee, alternating bilaterally.

CST training will take 60 minutes per day, starting with a brief warm-up programme to prepare the body, and ending with a cool-down programme. The entire intervention length is 5 days/week, totally 12 weeks.

#### 2.9.3 Tai Chi Chuan group

In addition to the routine activities of daily living, the participants in the TCC group will receive 12 weeks of TCC training. The TCC programme is composed of 24 movements from the Yang styles (http://www.egreenway.com/taichichuan/short.htm#List), which particularly involve deep breathing accompanied by the opening and closing of the hands and stepping forward and backward with weight transference.^[21]^ Before starting the TCC program, the participants will be instructed by certified TCC instructors, who will ensure that each participant is familiar with a standard TCC programme. TCC training will take 60 minutes per day; warm-up exercises of each session will be applied to the preparation of the neuromuscular system for the training loads, and sessions will end with a cool-down exercise to release muscle tension and stiffness (for example, arm swinging, simple stretching exercise, deep breathing, etc.).

The participants in the TCC training group will exercise for 5 days/week at the Ningxia Yuehai Service Center, for a total of 12 weeks.

#### 2.9.4 Combined training group

In addition to the routine activities of daily living, the participants in the CTG will receive 30 minutes CST and 30 minutes TCC training per day, 5 days/week, totally 12 weeks.

### 2.10 Follow-up period

During the 12 weeks follow-up period, all participants will be told to resume their original lifestyle without having to insist on special exercise intervention. In addition, if the participants cannot avoid an increase in exercise load, they will be required to record the details of the extra physical activity, including the type, intensity, and time. We will re-measure the primary outcomes at the end of the follow-up period.

### 2.11 Outcome assessment

The outcome assessment is mainly composed of three parts:

(i) musculoskeletal system index: bone mineral density, balance ability, flexibility, fall risk assessment, and so on;
(ii) cardiopulmonary function evaluation index: a step test, vital capacity, heart rate, and so on; and
(iii) other related indexes: activities of daily living, pain score, QOL, and so on.

All primary and secondary outcomes will be assessed by experienced therapists at the rehabilitation department of the People's Hospital of Ningxia Hui Autonomous Region at baseline and at 12 weeks (the end of the intervention). The process of the assessments is listed in Table 1.

### 2.12 Primary outcomes

#### 2.12.1 Bone mineral density (BMD)

We will assign a professional technician to perform standard BMD measurements by using DXA (GE Lunar iDXA with encore 12.0 software, GE Healthcare Lunar, Madison, WI).^[22]^ BMD measurements includes the whole body, lumbar spine (L1-L4), proximal femur of the left leg, and left fore-arm.^[23]^ In order to ensure the accuracy of the collected data, all instruments should be calibrated by the inspector before the measurement is carried out.

#### 2.12.2 Cardiopulmonary function

Cardiopulmonary function in this study will be evaluated by step test, vital capacity, blood pressure and heart rate. The step test will be carried out using a step tester (product type: CSTF-TZ-5000) manufactured by Beijing Titanium Oriental Co., Ltd. The vital capacity was measured using a vital capacity tester (product type: CSTF-FH-5000) produced by Beijing Zhongtai Oriental Co., Ltd. Blood pressure and resting heart rate will be tested by an electric sphygmomanometer produced by Omron, Dalian, China (product type: HEM-76C).

#### 2.12.3 Secondary outcomes

The balance ability will be measured by standing on the Pro-kin system (product type: PK254P; produced by Tecnobody S.r.l company, Italy) with eyes open for 30 seconds and eyes closed for 30 seconds. The Flexibility will be measured using Sit and Reach flexibility test equipment (product type: CSTF-TQ-5000, Zhongtitongfang Co., Ltd., Beijing, China). Fall risk will be assessed via the short-form Falls Efficacy Scale International.^[24]^ Activities of daily living will be assessed using the Chinese version of the Instrumental Activities of Daily Living (IADL).^[25]^We chose the VAS as the pain score, which is1 of the most commonly used pain intensity measurement (0∼4 mm can be regarded as no pain; 5∼44 mm represents mild pain; 45∼74 mm represents moderate pain; 75∼100 mm represents severe pain).^[26]^ QOL will be evaluated using the Chinese version of EQ-5D-5L.^[27]^

#### 2.12.4 Safety evaluation

Although there are few reports on the adverse events (AEs) of TCC exercise, any unexpected AEs during the intervention period such as lumbar sprain, knee or ankle sprain, and knee pain, etc. will be recorded in detail on a case report form (CRF). If serious AEs occur, the researchers will report immediately to the project leader and the ethics committee, and they will decide whether the participants need to withdraw from the research after negotiation.

#### 2.12.5 Data collection and management

After confirming that participants meet the inclusion and exclusion criteria, the research screeners will collect their demographic and baseline characteristics at the appropriate time. The primary and secondary outcomes will be measured by the outcome assessors at baseline and at 12 weeks (the end of the intervention). The primary outcome will be measured again at 25 weeks (during the follow-up period.)

The research assistant will complete the quality control of data collection and data entry. The research manager will complete the collection, classification, identification, coding, and analysis of raw data.

### 2.13 Statistical analysis

We will use the intention-to-treat (ITT) analysis and per-protocol subject (PPS) analysis to analyze the primary and secondary outcomes. When there is a missing data, we will use the last observation carried forward method to deal with it. The continuous variables will be expressed as the mean ± SD. All categorical variables will be expressed in terms of their standard error. After examining whether the data accord with normality test (the Kolmogorov–Smirnov test), we will use ANOVA (assuming a normal distribution) or Kruskal–Wallis (a nonparametric test) to analyze the measurement results. We will use the Bonferroni method to appropriately adjust the overall level of significance for multiple outcomes.

For all the analyses of this study a significance level of 95% (two-sided *P* < .05) will be used. All statistical tests will be conducted using SPSS Statistical Software (IBM SPSS Statistics for Windows, Version 21.0. Armonk, NY).

### 2.14 Quality control

To ensure that this trial can be completed with high quality, the researcher must strictly adhere to the requirements of the trial protocol, and the participants must be strictly in accordance with the inclusion and exclusion criteria. In addition, to ensure the authenticity and credibility of the data, we will advise participants that they are free to withdraw from the study at any stage for any reason without any consequences. Of course, if possible, we will try our best to persuade the participants to undergo the last evaluation before their withdraw. Thus, we can do an intention-to-treat (ITT) analysis. The participants must be grouped according to the random allocation sequence produced by the statistical software SAS 9.1. We stipulate that assessors and statistical analysts will not participate in the recruitment and allocation of participants.

### 2.15 Ethical consideration

This study protocol is in compliance with the requirements of the Helsinki declaration and has been approved by the medical ethics committee of the Ningxia Hui Autonomous Region People's Hospital, in China (Approval No. 2017-KY-003). All participants will be fully informed about the trial and will sign the written informed consent form before participation.

All participants in this study will be fully informed of the purpose and content of the study and sign written informed consent before participating in the study.

## 3 Discussion

The problem of aging has gradually become an international issue, but it is also a growing trend. As mentioned above, there may be some adverse consequences of aging related skeletal muscle dysfunction and multiple visceral disease in the elderly.^[3]^ More importantly, respiratory impairments and mobility restrictions often co-exist and may have a bi-directional association and similar impact on physical health, including exertional dyspnoea and subsequent disability and death.^[28,29]^ Due to the decline in many physical functions caused by age factors, a series of related strategies are needed to deal with aging, including social participation and maximizing community participation, which is a key component of positive aging.^[30,31]^ In view of this, the main criteria for the selection of exercise could be that it is enjoyable, targetedly improve different components of aerobic capacity, respiratory function and muscle strength, with a key objectives of motivating the majority of elderly people living in the community to participate regularly.^[32]^

As a low-medium-intensity aerobic exercise,^[33]^ TCC has been practiced for many centuries and is becoming more and more popular in the West.^[34]^ Compared to Tai Chi Chuan, core strength training is relatively new, but it is also a modern popular training method that is considered a feasible and safe way of training to produce significant health increases (i.e., strength, respiratory performance, activity ability) and skill related components (i.e., balance, coordination, agility)) in a healthy people.^[17,35]^ Based on previous clinical studies and systematic reviews, both TCC and CST have positive effects on human health outcomes.^[36–41]^ However, there is still lack of evidence that systematically compares the effects of TCC and CST on the musculoskeletal system and cardiopulmonary function in older adults, as well as the synergistic effect of a combination of the 2 physical training patterns.

Therefore, the main purpose of this trial is to solve the above 2 core issues. We carefully designed this randomized, single-blind, parallel-controlled trial to evaluate the effects of CST combined with TCC for musculoskeletal system and cardiopulmonary function in older adults. This trial consists of a 12-week intervention period and the use of standard musculoskeletal and cardiopulmonary function outcomes, including bone density detection, balance and co-ordination ability, walking ability, pain VAS score, fall risk assessment, activities of daily living, pulmonary function test, color sonography, and electrocardiogram.

### 3.1 Limitations

As with most clinical studies, there are also obvious limitations in this study. Ideally, all participants in clinical randomized controlled trials should follow the blind method, but this is difficult to achieve in non-pharmacological trials.^[42]^ Although it is impossible to conduct blinding of both the coaches and participants in this trial, we will reduce the possible bias by blinding the assessors and data analysts.

In summary, the results of this trial are expected to clarify the synergistic effect of CST and TCC training on musculoskeletal and cardiopulmonary function in older adults and to confirm whether combined or exclusive CST and TCC training, is more effective at improving functional outcomes in the elderly.

## Author contributions

LD C, XF J, and YZ conceived of the study, designed the study protocol, and drafted the manuscript. XF J, CJ wrote the manuscript. LD C is in charge of coordination and direct implementation. CJ, XF J, JT, and YY L helped to develop the study measures and analyses. All authors contributed to drafting the manuscript and have read and approved the final manuscript.

**Conceptualization:** cai jiang, jing tao, yinyan li, yu zhou.

**Data curation:** cai jiang, yinyan li, lidian chen.

**Formal analysis:** Xiaofei jia.

**Funding acquisition:** lidian chen, yu zhou.

**Methodology:** cai jiang, Xiaofei jia, jing tao, yinyan li.

**Project administration:** Xiaofei jia, lidian chen, yu zhou.

**Resources:** Xiaofei jia, lidian chen, yu zhou.

**Writing – original draft:** cai jiang, Xiaofei jia, yinyan li.

**Writing – review & editing:** cai jiang, jing tao, lidian chen.
